# Developing new age-specific prostate-specific antigen thresholds for testing for prostate cancer

**DOI:** 10.1007/s10552-018-1014-3

**Published:** 2018-02-16

**Authors:** Rebecca Gilbert, Kate Tilling, Richard M. Martin, J. Athene Lane, Michael Davis, Freddie C. Hamdy, David E. Neal, Jenny L. Donovan, Chris Metcalfe

**Affiliations:** 1 Population Health Sciences, Bristol Medical School, University of Bristol, 39 Whatley Road, Bristol, BS8 2PS UK; 2 MRC Integrative Epidemiology Unit, University of Bristol, Oakfield House, Oakfield Grove, Bristol, BS8 2BN UK; 3 Nuffield Department of Surgical Sciences, University of Oxford, John Radcliffe Hospital, Headington, Oxford, OX3 9DU UK; 4 Department of Oncology, University of Cambridge, Addenbrook’s Hospital, Hills Road, Cambridge, CB2 0QQ UK

**Keywords:** Prostate cancer, Prostate-specific antigen testing, Biopsy, Age, Reference ranges

## Abstract

**Purpose:**

To examine whether age-related reference ranges for “normal” prostate-specific antigen (PSA) change (determined in men without prostate cancer) can be used to identify men at high risk of having prostate cancer.

**Methods:**

Subjects were men aged 50–69 years with PSA < 10 ng/mL from the UK-based Prostate Testing for cancer and Treatment (ProtecT) study. Men with prostate cancer were categorized as high or low risk of progression (Low risk: Gleason score ≤ 6 and stage T1–T2a; High risk: Gleason score 7–10 or stage T2C). Men without prostate cancer were those with no histological confirmation of prostate cancer. Previously developed longitudinal reference ranges for normal age-related PSA change were used to calculate an age-specific PSA threshold. We compared the ability of our age-specific PSA threshold to discriminate between high- and no/low-risk prostate cancer with that of two existing thresholds: (i) threshold of PSA = 3 ng/ml for all ages; (ii) National Institute of Clinical Excellence (NICE) guidelines dependent on age-group thresholds (age 50–59: PSA = 3 ng/mL; age 60–70: PSA = 4 ng/mL; age ≥ 70: PSA = 5 ng/mL).

**Results:**

We included 823 men with high-risk prostate cancer and 80,721 men with no/low-risk prostate cancer. A threshold of PSA = 3 ng/ml for all ages identified more high-risk prostate cancers, recommending biopsy in 9.8% of men, of which 10.3% (*n* = 823) had high-risk prostate cancer. Using the NICE guidelines as the threshold for biopsy, 6.9% men were recommended for biopsy, of which 11.9% (*n* = 668) had high-risk prostate cancer. Using the new age-specific threshold for biopsy, 2.3% men were recommended for biopsy, of which 15.2% (*n* = 290) had high-risk prostate cancer. The age-specific threshold identified fewer high-risk prostate cancers, but fewer men received unnecessary biopsy.

**Conclusion:**

There is no benefit to using reference ranges for “normal” PSA that change with age nor the age-specific thresholds suggested by the NICE guidelines. While the age-varying thresholds are more discriminatory, too many high-risk cancers are missed.

**Electronic supplementary material:**

The online version of this article (10.1007/s10552-018-1014-3) contains supplementary material, which is available to authorized users.

## Introduction

Prostate-specific antigen (PSA) testing, followed by biopsy if the PSA level is raised (typically ≥ PSA 3–4 ng/mL), is a widely accepted screening method for prostate cancer [1]. However, most screen-detected prostate cancers have low risk of progression, with potential harm caused by unnecessary treatment [2, 3].

Despite widespread use of PSA testing, men with raised PSA may have no evidence of prostate cancer at biopsy, while not all men with prostate cancer have raised PSA [4]. PSA levels increase with age, and the natural variability in PSA level (both within men over time, and between men) is likely to be greater in older men [5], thus obscuring disease-related changes. Current age-related PSA thresholds are based on cross-sectional data and hence do not attempt to distinguish these different sources of variability, nor to describe serial changes in PSA level for aging individuals.

The aim of the current study was to examine whether age-related reference ranges for “normal” PSA change (determined in men without prostate cancer) can be used to identify men at high risk of having prostate cancer. We hypothesize that a higher threshold will be identified for older men, which will identify clinically relevant prostate cancers at high risk of progression while saving some men from unnecessary biopsy.

## Methods

The study is nested within a multicenter randomized controlled trial of treatments for localized prostate cancer: the Prostate Testing for cancer and Treatment (ProtecT) study [6]. Between 2001 and 2009, over 100,000 men aged 50–69 years attended a ‘prostate check clinic’ where they were offered a PSA test. Those with raised levels (≥ 3 ng/mL) were offered diagnostic biopsy. Tumors were histologically confirmed, clinically staged (“localized”: T1–T2; “locally advanced”: T3–T4) [7], and Gleason graded.

Men were included in the current study if they had PSA < 10 ng/mL, as PSA above this level would normally warrant further investigation.

Men with prostate cancer were categorized as low risk of progression if their Gleason score was six or less and stage T1–T2a, and as high risk of progression if their Gleason score was seven to ten or stage T2b–T4, adapted from the NICE guidelines [9]. For 137 men who had Gleason score ≤ 6 but no stage, and 23 men who had stage T1–T2a but no Gleason score, a low risk of progression was assumed.

Men without prostate cancer were defined as those with no histological confirmation of prostate cancer, i.e., (i) PSA < 3 ng/mL; (ii) PSA ≥ 3 ng/mL and a negative biopsy result.

Longitudinal reference ranges for normal PSA change with age were developed previously using data from the Krimpen longitudinal community-based study [8], specifically serial PSA measurements from men aged 50–78 years who did not have prostate cancer (*n* = 1,462).

**Fig. 1:**
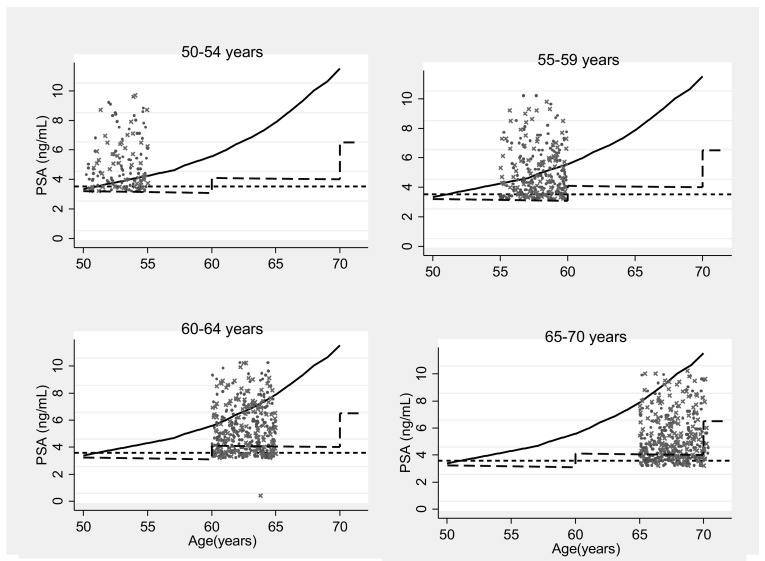
Graph showing PSA level (ng/mL) plotted against age (both measured at the prostate check clinic).The lines depict the three thresholds of PSA which may trigger further investigations (i) a threshold of PSA = 3 ng/mL for all ages (--- line); (ii) NICE thresholds (age 50–59: PSA = 3 ng/mL; age 60–70: PSA = 4 ng/mL; age ≥ 70: PSA = 5 ng/mL) (-- -- -- line); (iii) age-specific threshold developed in the current study above which the fastest increasing 2.5% of men lie (solid line). The performance of the threshold depends on the age distribution of the data so the graphs have been stratified by age group. A random sample of men were plotted to improve readability. X indicates a man diagnosed with clinically significant prostate cancer (1 in 200 men plotted, *n* = 406). A dot indicates a man not diagnosed with prostate cancer or with prostate cancer at low risk of progression (1 in 100 men, *n* = 813). An upper bound for age-specific PSA level, above which the fastest increasing 2.5% of men lie, can be estimated using the PSA level for each age as described by the Krimpen study [8]. The plotted points, where age (years):PSA(ng/mL), are: 50:2.8, 51:3.0, 52:3.2, 53:3.4, 54:3.6, 55:3.8, 56:4, 57:4.2, 58:4.6, 59:4.9, 60:5.2, 61:5.6, 62:6.1, 63:6.5, 64:7, 65:7.6, 66:8.3, 67:9, 68:9.8, 69:10.4, 70:11.3

An upper bound for age-specific PSA level, above which the fastest increasing 2.5% of men lie, can be estimated using the PSA level for each age as described by Krimpen (Fig. 1). If PSA falls above this reference range, then it is implied that the patient’s PSA is above what would be expected due to normal aging. For example, at age 50, a man would be recommended for biopsy if his PSA ≥ 2.8 ng/mL. At age 55 years, a man would be recommended for biopsy if his PSA ≥ 3.8 ng/mL, and, at 65 years, if his PSA ≥ 7.6 ng/mL.

We compared the ability of the new age-specific threshold to discriminate between high-risk and no/low-risk prostate cancer with that of two existing thresholds: (i) threshold of PSA = 3 ng/ml for all ages; (ii) National Institute for Health and Care Excellence (NICE) guidelines depending on age group (age 50–59: PSA = 3 ng/mL; age 60–70: PSA = 4 ng/mL; age ≥ 70: PSA = 5 ng/mL) [9].

For each threshold, we calculated the number of (i) men with high-risk prostate cancer and PSA above the threshold (i.e., true positive (TP)), (ii) men with no or low-risk prostate cancer and PSA below the threshold (i.e., true negative (TN)), (iii) men with no or low-risk prostate cancer and PSA above the threshold (i.e., false positive (FP)), and (iv) men with high-risk prostate cancer and PSA below the threshold (i.e., false negative (FN)). Analyses are stratified by age group.

We additionally calculated the diagnostic likelihood ratio (LR) as sensitivity/(1-specificity), where sensitivity is the proportion of high-risk prostate cancers correctly identified as such (= TP/(TP + FN)) and specificity is the proportion of no or low-risk prostate cancers correctly identified as such (TN/(FP + TN)). A higher value of the LR indicates that a test is better able to discriminate between men with high-risk prostate cancer and those with no or low-risk prostate cancer.

Analyses were carried out in Stata 14 (StataCorp, 2014. College Station, TX).

## Results

There were 81,553 men aged ≥ 50 years with at least one PSA result and PSA ≤ 10 ng/mL. 9 men were dropped from analysis as their clinical information was missing. Of the remaining 81,544 men, 2,556 (3.1%) men had prostate cancer. The current analysis includes 823 men with clinically relevant prostate cancer at high risk of progression and 80,721 men with no (*n* = 78,988) or low risk of progression (*n* = 1,733) prostate cancer.

There were no substantial differences in baseline characteristics between men with high-risk prostate cancer and no/low-risk prostate cancer other than mean age (62.4 years vs. 59.3 years, *p* ≤ 0.001) and mean PSA level (5.5 vs. 1.3 ng/mL, *p* ≤ 0.001) (Table S1).

Using a threshold of PSA = 3 ng/ml for all ages as the threshold for biopsy resulted in 8,015 men (9.8%) being recommended for biopsy, of which 823 (10.3%) had high-risk prostate cancer. THE LR was 11.2 (Table 1; Fig. 1).

**Table 1:** Comparison of performance of the three thresholds for PSA (i) threshold of PSA = 3 ng/ml for all ages; (ii) NICE thresholds (age 50–59: PSA = 3 ng/mL; age 60–70: PSA = 4 ng/mL; age ≥ 70: PSA = 5 ng/mL); (iii) age-specific threshold developed in the current study above which the fastest increasing 2.5% of men lie

	Men with high-risk prostate cancer, PSA above threshold, i.e., true positives (*n*)	Men with high-risk prostate cancer, PSA below threshold, i.e., false negatives (*n*)	Proportion of high-risk prostate cancer correctly identified as such^a^ (95% CI)	Men with no/low-risk prostate cancer, PSA below threshold, i.e., true negatives (*n*)	Men with no/low-risk prostate cancer, PSA above threshold, i.e., false positives (*n*)	Proportion of no or low-risk prostate cancer correctly identified as such^a^ (95% CI)
All Men						
New age-specific PSA threshold	290	533	35.2 (32.0, 38.6)	79,102	1,619	98.0 (97.9, 98.1)
NICE guidelines	668	155	81.2 (78.3, 83.8)	75,763	4,958	93.9 (93.7, 94.0)
PSA = 3 ng/mL	822	1^b^	99.9 (99.3, 100.0)	73,528	7,193	91.1 (90.9, 91.3)
Age group 50–54 years						
New age-specific PSA threshold	73	11	86.9 (77.8, 93.3)	20,759	572	97.3 (97.1, 97.5)
NICE guidelines	84	0	100.0 (95.7,100.0)	20,558	773	96.4 (96.1, 96.6)
PSA = 3 ng/mL	84	0	100.0 (95.7, 100.0)	20,558	773	96.4 (96.1, 96.6)
Age group 55–59 years						
New age-specific PSA threshold	111	66	62.7 (55.1, 69.9)	23,973	632	97.4 (97.2, 97.6)
NICE guidelines	177	0	100.0 (97.9, 100.0)	22,904	1,701	93.1 (92.8, 93.4)
PSA = 3 ng/mL	177	0	100.0 (97.9, 100.0)	22,904	1,701	93.1 (92.8, 93.4)
Age group 60–64 years						
New age-specific PSA threshold	86	174	33.1 (27.4, 39.2)	19,575	362	98.2 (98.0, 98.4)
NICE guidelines	181	79	69.6 (63.6, 75.1)	18,692	1,245	93.8 (93.4, 94.1)
PSA = 3 ng/mL	259	1	99.6 (97.9, 100.0)	17,579	2,358	88.2 (87.7, 88.6)
Age group ≥ 65 years						
New age-specific PSA threshold	20	282	6.6 (4.1, 10.0)	14,795	53	99.6 (99.5, 99.7)
NICE guidelines	226	76	74.8 (69.5, 79.6)	13,609	1,239	91.7 (91.2, 92.1)
PSA = 3 ng/mL	302	0	100.0 (98.8, 100.0)	12,487	2,361	84.1 (83.5, 84.7)

^a^Equivalent to internal estimates of sensitivity = TP/(TP + FN) and specificity = TN/(FP + TN) where TP is true positives, FN is false negatives, TN is true negatives, FP is false positives

^b^One man had PSA = 0.2 at the prostate check clinic, and received a biopsy which identified prostate cancer

Using the NICE guidelines based on age group as the threshold for biopsy resulted in 5,626 (6.9%) men being recommended for biopsy, of which 668 (11.9%) had high-risk prostate cancer. Within men who had no or low-risk prostate cancer 2,235 men were saved unnecessary biopsy when using the NICE guidelines based on age group compared with a threshold of PSA = 3 ng/mL for all ages at the cost of not identifying 155 men with high-risk prostate cancer. The LR was 13.3 (Table 1; Fig. 1).

Using the new age-specific threshold for biopsy resulted in 1,909 (2.3%) men being recommended for biopsy, of which 290 (15.2%) had high-risk prostate cancer. Within men who had no or low-risk prostate cancer 5,579 men were saved unnecessary biopsy when using the new age-specific threshold compared with a threshold of PSA = 3 ng/mL for all ages at the cost of not identifying 533 men with high-risk prostate cancer. The LR was 17.6 (Table 1; Fig. 1).

## Discussion

In UK men aged 50–69 years, using reference ranges for “normal” PSA change with age or the age group-specific thresholds suggested by the NICE guidelines resulted in fewer unnecessary biopsies but at the cost of more missed prostate cancers. While the threshold of PSA = 3 ng/mL for all ages identified more prostate cancers at high risk of progression than either of the other two thresholds, resulting in fewer missed prostate cancers, more men received an unnecessary prostate biopsy. The diagnostic likelihood ratios suggest that the thresholds that incorporate age do give a more discriminatory test. However, the number of high-risk prostate cancers that are missed by the age-specific threshold test and the thresholds suggested by the NICE guidelines means that these tests are unacceptable in practice.

Other age-adjusted PSA thresholds for biopsy have been suggested [10], although these have been for age groups and based on cross-sectional data. Tests of these age-adjusted cut-offs showed inconclusive results, although age-adjusted thresholds for PSA were recommended in the majority of cases. This method has not become widely accepted in clinical use or screening programs among concerns about missing a high proportion of clinically significant cancers in older men while augmenting the rate of unnecessary biopsies in younger men. Results from the Tyrol Prostate Cancer Early Detection Program found that age-adjusted PSA thresholds using PSA and free PSA levels achieved a similar sensitivity while simultaneously reducing the number of biopsies [11]. Results from another study using the same data as the current study found that an age- and BMI-adjusted PSA model was no more clinically useful for detecting prostate cancer than the current NICE guidelines [12].

Men in the ProtecT study with a PSA < 3 ng/mL were not biopsied and may have had undiagnosed prostate cancer, resulting in calculated sensitivities that are not reflective of the true sensitivities. Consequently, while the sensitivity and specificity for the PSA thresholds in this study can be directly compared, they should not be compared with the true sensitivity and specificity of PSA testing as published by previous studies, and we do not refer to them as sensitivity or specificity throughout this article to avoid confusion. The new age-specific threshold would recommend that men aged 50 years use a threshold of PSA = 2.8 ng/mL. These men were not biopsied in ProtecT and so may have had undiagnosed prostate cancer.

The only threshold that would lead to men with a PSA < 3 ng/mL being biopsied is the new age-specific threshold, where the threshold is less than 3 ng/mL for younger men (men aged 50 years would be biopsied if their PSA was 2.8 ng/mL). By the time the men are 51 years, the threshold is 3 ng/mL.

## Conclusion

In this cohort of UK men aged 50–69 years, there is no evidence of benefit from using reference ranges for “normal” PSA change with age nor the age-specific thresholds suggested by the NICE guidelines (age 50–59: PSA = 3 ng/mL; age 60–70: PSA = 4 ng/mL; age ≥ 70: PSA = 5 ng/mL). A threshold of PSA = 3 ng/mL for all ages identified more clinically relevant prostate cancers at high risk of progression than either of the other two thresholds, resulting in fewer missed prostate cancers, but at the cost of more men receiving an unnecessary prostate biopsy. While the age-varying thresholds are more discriminatory, too many high-risk cancers are missed.

## Electronic supplementary material

Below is the link to the electronic supplementary material.

Supplementary material 1 (XLSX 12 KB)
